
JOURNAL INFORMATION
==============================
Journal ID: Wirtschaftsdienst

Wirtschaftsdienst

EISSN: 1613-978X

ARTICLE INFORMATION
==============================
PMCID: PMC9483380
DOI: 10.1007/s10273-022-3276-3
Article ID: 3276
Article version: 1
Subjects: Zeitgespräch

Die Zeitenwende erreicht den deutschen Arbeitsmarkt

Germany’s Labour Market at a Turning Point

Bonin Holger bonin@iza.org
1
Rinne Ulf rinne@iza.org
2
1 grid.424879.4 0000 0001 1010 4418 Forschungsinstitut zur Zukunft der Arbeit (IZA), Schaumburg-Lippe-Straße 5-9, 53113 Bonn, Deutschland
2 grid.424879.4 0000 0001 1010 4418 Scientific Management/Senior Research Associate, Forschungsinstitut zur Zukunft der Arbeit (IZA), Schaumburg-Lippe-Strasse 5-9, 53113 Bonn, Deutschland
Electronic publication date: 2022 Sep 16
Print publication date: 2022
Volume: 102
Issue: 9
First page: 665
Last page: 668
Copyright: © Der/die Autor:in 2022
License: Open Access: Dieser Artikel wird unter der Creative Commons Namensnennung 4.0 International Lizenz veröffentlicht (https://creativecommons.org/licenses/by/4.0/deed.de).
Open Access wird durch die ZBW — Leibniz-Informationszentrum Wirtschaft gefördert.
License URL: https://creativecommons.org/licenses/by/4.0/

Keywords: J01, J18, J21, J68
issue-copyright-statement: © The Author(s) 2022

==============================
The COVID-19 pandemic and the Ukraine war mark a turning point for the German labour market. These crises accelerate transformative forces that have been at work for some time, such as digitalisation and decarbonisation, and are likely to permanently change the international division of labour and mobility. The shortage of skilled workers, which already hampered development ahead of the crisis, has now grown into a broader labour shortage and has also reached the low-wage sector. This article outlines how labour shortages could be countered by concerted action from the supply and demand sides. It shows that coping with the changes in the German labour market requires more efforts from policymakers, firms and the labour force.

Prof. Dr. Holger Bonin ist Forschungsdirektor des IZA — Institut zur Zukunft der Arbeit in Bonn und lehrt Volkswirtschaftslehre mit den Schwerpunkten Arbeitsmarkt- und Sozialpolitik an der Universität Kassel.

Bundeskanzler Olaf Scholz sprach in seiner Rede am 24. Februar 2022 im Deutschen Bundestag angesichts des russischen Überfalls auf die Ukraine von einer geschichtlichen Zeitenwende (Deutscher Bundestag, 2022). Auch für den deutschen Arbeitsmarkt könnte der Ukrainekrieg – gemeinsam mit der noch nicht endgültig beendeten Coronapandemie – eine Zäsur markieren: als Beschleuniger bereits länger wirkender transformativer Kräfte, wie Dekarbonisierung und Digitalisierung, und als Auslöser nachhaltiger Veränderungen in der internationalen Arbeitsteilung. Der Fachkräftemangel, der schon vor der Krise die Entwicklung der deutschen Wirtschaft hemmte, hat sich zu einem Arbeitskräftemangel ausgewachsen, der auch den Niedriglohnsektor erreicht hat.

## Deutscher Arbeitsmarkt in Zeiten von Pandemie und Ukrainekrieg

Im Frühjahr 2020 schlitterte der deutsche Arbeitsmarkt schwächelnd in die Pandemie, hat sich dann aber im Verlauf der Coronakrise erneut bemerkenswert robust gezeigt. Die Beschäftigung konnte, etwa durch den Einsatz von Kurzarbeit, erfolgreich stabilisiert werden (Bonin et al., 2021a). Dies geschah allerdings auf Kosten einer deutlich reduzierten Arbeitsmarktdynamik, mit nur wenigen krisenbedingten Entlassungen und zugleich merklich weniger Neueinstellungen. Die Folgen davon machen sich derzeit deutlich bemerkbar. Denn jeder Versuch, den Pausenmodus auf dem Arbeitsmarkt zu betätigen und den Status quo zu zementieren, ist am Ende zum Scheitern verurteilt. Die Arbeitswelt wandelt sich trotzdem – auch krisenbedingt.

So wirkte die Pandemie in vielen Bereichen wie ein Katalysator, etwa indem sich Prozesse der Digitalisierung beschleunigten und tradierte Muster der Präsenzarbeit zugunsten von Homeoffice und mobiler Arbeit über Bord geworfen wurden (Bonin et al., 2021b). Aufgrund ausgebliebener Neueinstellungen fehlen derzeit Arbeitskräfte vor allem in besonders von der Coronakrise betroffenen Branchen, die ohnehin eine hohe Personalfluktuation aufweisen und über längere Zeit kaum neu eingestellt haben (Röttger und Weber, 2022). Der Wanderungssaldo fiel in der Pandemie auf ein geringes Niveau und erholt sich nur langsam. Insbesondere die EU-Binnenmigration kann daher Personallücken nicht in dem Maße auffangen, wie dies in den Zeiten des deutschen „Arbeitsmarktwunders“ während der 2010er Jahre der Fall war.

Die Auswirkungen der Coronakrise haben die Abhängigkeit des deutschen Arbeitsmarkts von einem funktionierenden Niedriglohnsektor und ständiger Arbeitskräftezuwanderung verdeutlicht – und die damit verbundenen Risiken.

Zudem führen die Auswirkungen von Pandemie und Ukrainekrieg die Abhängigkeit des deutschen Modells von globalen Lieferketten und dem preiswerten Import von Rohstoffen vor Augen. Die bereits zuvor geführte Debatte um einen Rückzug aus der Globalisierung (Straubhaar et al., 2021), Schlüsseltechnologien und technologische Souveränität (EFI, 2022) hat deshalb an Intensität zugenommen. Außerdem gibt es einen erheblichen Schub für die Energiewende und die Dekarbonisierung der Gesellschaft. Dies hat erhebliche Implikationen für die Nachfrage nach Arbeitskräften. So würden, um die Klimaziele der Bundesregierung in der energetischen Gebäudesanierung bis 2030 umzusetzen, etwa die Installation von sechs Mio. Wärmepumpen, zusätzlich 190.000 Fachkräfte im Handwerk gebraucht (IG Metall et al., 2022). Durch den Ukrainekrieg müssten diese Wärmepumpen sogar noch schneller installiert werden – eine angesichts von Lieferengpässen und Personalmangel kaum zu bewältigende Herausforderung.

## Arbeitsmarktengpässe als gesellschaftliche Herausforderung

Die krisenbedingten Engpasslagen werden noch dadurch verstärkt, dass sich jetzt der demografische Schock des Eintritts der Babyboomer-Generation in den Ruhestand sehr schnell entfaltet. Den sich daraus ergebenden enormen Ersatzbedarf können die neu in den Arbeitsmarkt eintretenden Jahrgänge, auch wenn sie im Durchschnitt besser beruflich qualifiziert sind als die altersbedingt ausscheidenden Jahrgänge, zahlenmäßig nicht ausgleichen. So drohen nicht nur bei Fachkräften erhebliche Engpässe,

**Prof. Dr. Holger Bonin** ist Forschungsdirektor des IZA – Institut zur Zukunft der Arbeit in Bonn und lehrt Volkswirtschaftslehre mit den Schwerpunkten Arbeitsmarkt- und Sozialpolitik an der Universität Kassel.

sondern auch bei Arbeitskräften im Bereich von Tätigkeiten mit geringerem Anforderungsniveau. Schon heute fehlen z. B. in Deutschland bis zu 80.000 Lkw-Fahrer:innen – und jährlich steigt diese Zahl um knapp 15.000 Personen, weil der altersbedingte Ersatzbedarf von 30.000 Personen nicht gedeckt werden kann (BGL, 2021).

Die Engpässe am deutschen Arbeitsmarkt sind aus volkswirtschaftlicher Sicht mit erheblichen Kosten verbunden (Bonin, 2020). Um Wachstumsklemmen zu lösen und das für eine erfolgreiche Bewältigung der akuten transformativen Prozesse erforderliche Humankapital zu sichern, sind Anstrengungen aller gesellschaftlichen Akteur:innen gefordert. Die Arbeitgebenden müssen sich noch aktiver um die Gestaltung attraktiver Arbeitsplätze sowie die Aus- und Weiterbildung von Personal bemühen. Beschäftigte und Arbeitslose sind gefordert, zusätzliche und gänzlich neue berufliche Qualifikationen zu erwerben. Auf sie könnten auch längere effektive Arbeitszeiten zukommen – in der Woche, im Jahr oder über das gesamte Erwerbsleben –, um sinkende Pro-Kopf-Einkommen und damit einen Rückgang des Wohlstandsniveaus im demografischen Wandel zu verhindern. Schließlich ist der Staat gefragt: Primär muss er die für eine hohe Anpassungsbereitschaft und Anpassungsfähigkeit der Akteur:innen auf beiden Marktseiten nötigen Rahmenbedingungen setzen. Darüber hinaus sollte er da korrigierend eingreifen, wo Unvollkommenheiten (wie Informationsmängel) oder Externalitäten (wie Skaleneffekte) gesamtwirtschaftlich effiziente Lösungen der privaten Akteur:innen verhindern.

## Arbeitsbedingungen verbessern

Wie bedeutsam gute Arbeitsbedingungen und die Aufwertung von Tätigkeiten – in erster Linie in der Verantwortung der Arbeitgebenden liegende Faktoren – zur Vermeidung von Arbeitskräfteengpässen sind, zeigt sich aktuell in Teilen des Niedriglohnsektors. So fehlen in der Abfertigung an den Flughäfen oder in der Gastronomie akut Beschäftigte. In diesen Bereichen wurde in der Coronakrise statt auf Kurzarbeit vielfach auf „Fire and Re-Hire" gesetzt, ohne zu berücksichtigen, dass viele Arbeitskräfte die Gelegenheit nutzen könnten, sich nach attraktiveren Jobs umzusehen oder in ihre Herkunftsländer zurückzuwandern. Bei extrem angespannter Personalsituation und damit steigender Arbeitsbelastung droht nun ein Teufelskreis. Um zu verhindern, dass noch mehr Arbeitskräfte die betroffenen Arbeitsmarktsegmente verlassen, erscheint eine rasche Verbesserung der Arbeitsbedingungen zentral – und hier wiederum der Lohn als wichtigste und auch kurzfristig zu drehende Stellschraube. Dabei könnten firmenübergreifende Lösungen, etwa mittels Einbeziehung der Sozialpartner, Trittbrettfahrerverhalten vermeiden. Aber auch nicht monetäre Elemente, wie Unterstützung der Work-Life-Balance und berufliche Aufstiegsmöglichkeiten, könnten einem fehlenden Arbeitskräfteangebot entgegenwirken. Vor allem im Niedriglohnsektor stellt sich angesichts der inhärent hohen wirtschaftlichen Risiken für die dort Beschäftigten aus gesellschaftlicher Sicht jedoch die Frage, ob Personallücken zumindest mittelfristig nicht besser durch arbeitssparenden technischen Fortschritt, also von der Nachfrageseite her, beseitigt werden sollten. Die berufliche Tätigkeit der noch verbleibenden Beschäftigten würde dann durch einen höheren Anteil von Nicht-Routinetätigkeiten eine Aufwertung erfahren und – gegebenenfalls nach entsprechender Weiterbildung – höhere Löhne rechtfertigen.

## Zuwanderungshürden angehen

Als schon kurzfristig wirkendes Mittel gegen Engpässe am Arbeitsmarkt kommt auch Zuwanderung infrage. Auch deswegen haben Geflüchtete aus der Ukraine schnell einen stark erleichterten Zugang zum deutschen Arbeitsmarkt erhalten. Schon im Mai 2022 hatten 24 % der Betriebe im Gastgewerbe Kontakt zu Arbeitsuchenden aus der Ukraine – weit mehr als in von fehlenden Arbeitskräften weniger betroffenen Branchen (Gleiser et al., 2022). Generell macht Deutschland inzwischen großzügige Zuwanderungsangebote für Fachkräfte aus Drittstaaten, die aber nur auf geringe Resonanz stoßen. Verantwortlich hierfür sind speziell die Passivität bei der Zielgruppenansprache, ein weiterhin komplexes System von Zuwanderungsbedingungen in Kombination mit der Sprachbarriere, sowie die weiterhin große Zögerlichkeit bei der Anerkennung von im Ausland erworbenen Qualifikationen. Bisher ist unklar, ob die EU-Binnenmigration nach Deutschland wieder auf das Niveau vor der Coronapandemie zurückkehren wird – und diese Defizite wie zuvor ein gutes Stück weit kaschieren kann.

Damit aus guten Zuwanderungsangeboten für Fachkräfte auch genug Fachkräfteeinwanderung wird, müsste ein kohärentes Gesamtpaket von Maßnahmen geschnürt werden. Dabei sollte nicht nur die Einreise in den Blick genommen werden, sondern auch die Phase davor und ein Zeitraum danach. Außerdem müssen zwei fundamentale Probleme adressiert werden. *Erstens* ist die deutsche Sprache eine hohe Hürde und im internationalen Wettbewerb ein Standortnachteil. Dieser lässt sich nicht vollständig ausgleichen, aber etwa durch fremd- und vor allem englischsprachige Alltags-, Kultur- und Freizeitangebote reduzieren. Auch sollte das Angebot von berufsbezogenen Sprachkursen im Ausland weiter ausgebaut werden – eine Aufgabe für die Goethe-Institute. *Zweitens* müssen die Verfahren zur Anerkennung von Berufsabschlüssen schneller, digitaler und pragmatischer werden. Hierbei können die Erfahrungen mit der Kompetenzfeststellung und der Anerkennung von Teilqualifikationen im Kontext der Fluchtmigration hilfreich sein. Statt nach formalen Zertifikaten – übersetzt auf Deutsch und in Papierform – zu fragen, muss es mehr darauf ankommen, was Zugewanderte in der beruflichen Praxis können. Das festzustellen ist aber, wie bei Bewerbungen aus Deutschland nicht anders, primär Aufgabe der Arbeitgebenden.

## Inländisches Arbeitskräfteangebot stärken

Auch wenn Erwerbsmigration gewiss einen zentralen Baustein zur Sicherung der Fachkräftebasis darstellt, kann Deutschland sich nicht allein darauf verlassen. Der demografische Wandel betrifft viele Ziel- und Herkunftsländer ähnlich, sodass auch der Markt für international mobile gesuchte Fachkräfte zunehmend enger wird. Darum gilt es auch, noch vorhandene inländische Arbeitskräftepotenziale möglichst voll auszuschöpfen.

Nach wie vor erschwert es das deutsche Steuer- und Abgabensystem, dass Teilzeitbeschäftigte – insbesondere Mütter – ihren oft vorhandenen Wunsch nach einer längeren Arbeitszeit verwirklichen. Eine solche Hürde ist an erster Stelle die steuer- und sozialrechtliche Sonderbehandlung der geringfügig entlohnten Beschäftigung. Die Minijobs erweisen sich häufig als karriereschädliche Teilzeitfallen ohne Brückenfunktion in reguläre Beschäftigung und leisten zudem Dequalifizierung Vorschub (Wippermann, 2012). Sie drücken damit sowohl die Quantität als auch die Qualität des Arbeitsangebots. Die Sonderstellung geringfügiger Beschäftigung sollte deshalb begrenzt werden, etwa durch eine Beschränkung auf bestimmte Personengruppen wie Studierende und Personen im Ruhestand, oder durch eine generelle Einbeziehung in das der Lohn- und Einkommensteuer unterliegende Einkommen (Bonin et al., 2021c). Dieses Vorgehen trüge auch zu den oben skizzierten Zielen bei, den Niedriglohnsektor zu verkleinern und einen Wandel hin zu höherwertigen Arbeitsplätzen in den betroffenen Branchen anzustoßen. Darum erscheint die Anhebung der Minijob-Verdienstgrenze zum 1. Oktober 2022 mit Blick auf eine nachhaltige Überwindung von Arbeitskräfteengpässen kontraproduktiv. Sie dürfte die Teilzeitfalle durch geringfügige Beschäftigung noch verschärfen (Blömer und Consiglio, 2022).

Bei Verheirateten begünstigt zudem das Ehegattensplitting in Kombination mit der Steuerklassenkombination III-V ein Erwerbsmodell, bei dem in der Regel der Partner in Vollzeit arbeitet und die Partnerin in Teilzeit hinzuverdient. Bereits die laufende Besteuerung grundsätzlich nach Steuerklassenkombination IV-IV könnte durch ein geändertes Framing die Arbeitsanreize in der Zweitverdienendenposition stärken, ohne die tatsächliche Steuerlast der Haushalte zu verändern. Ein Übergang zu Alternativen, die der verfassungsrechtlichen Vorgabe der Nichtschlechterstellung von Verheirateten gegenüber Unverheirateten gerecht werden, wie ein Realsplitting oder eine Individualbesteuerung, würde die Steuerlast von Verheirateten gegenüber dem Status quo dagegen erhöhen. Dies ist politisch unpopulär – würde aber, wie Simulationsstudien zeigen, das Arbeitsangebotsvolumen insgesamt erhöhen (Bachmann et al., 2021; Blömer et al., 2021).

Um die insbesondere von Müttern angestrebten Tätigkeiten in langer Teilzeit bzw. kurzer Vollzeit zu ermöglichen, muss auch die Betreuungsinfrastruktur weiter verbessert werden. Der Mitte der 2000er Jahre einsetzende Ausbautrend hat die Integration von Müttern in den deutschen Arbeitsmarkt spürbar erhöht. Allerdings liegt Deutschland, was die Versorgung mit guter, verlässlicher und von den Betreuungszeiten her flexibler Kinderbetreuung angeht, in vielen Regionen immer noch spürbar hinter skandinavischen Vorbildern (Dänemark, Norwegen, Schweden) zurück. Dabei entspräche eine quantitative und qualitative Aufstellung der Kinderbetreuung wie dort den Wünschen vieler heutiger Eltern (OECD, 2017).

## Aus- und Weiterbildungssystem leistungsfähiger machen

Schweren gesellschaftlich-ökonomischen Krisen ist, wie transformativen Prozessen, qualifikatorischer Mismatch inhärent. Ihr disruptiver Charakter zerstört in manchen Teilen der Wirtschaft Arbeitsplätze, während mit den Anpassungen zur Bewältigung der großen Herausforderungen in anderen Teilen neue Jobs entstehen. Unterscheiden sich die Qualifikationsanforderungen in den betroffenen Bereichen, kann der Aufbau der neuen Jobs ins Stocken geraten, weil die vorhandenen Arbeitsuchenden dafür nicht ohne Weiteres infrage kommen. Die Überwindung Mismatch-bedingter Fachkräfteengpässe erfordert Zeit – und ein leistungsfähiges Aus- und Weiterbildungssystem.

Die duale Ausbildung ist traditionell eine Stärke Deutschlands. Sie hat in den Augen vieler Jugendlicher aber offenbar an Glanz verloren. Wir sehen einen Run an Hochschulen und berufliche Schulen aller Art – auch von jungen Menschen, die im klassischen System der beruflichen Bildung besser aufgehoben wären. Darum braucht es mehr Berufsorientierung, die Chancen und ein modernes Bild der Ausbildungsberufe vermittelt, etwa in den zur Bewältigung der Energiewende essenziellen Handwerken. Doch Information allein reicht natürlich nicht, auch die Praxis muss attraktiv sein. So ist eine Qualitätsoffensive zur Digitalisierung und für besseren Unterricht an den Berufsschulen dringend geboten. Ausbildungsinhalte, aber auch die Personalführung des in den Betrieben ausgebildeten Nachwuchses müssen mehr Flexibilität und Autonomie zulassen.

Im Bereich der beruflichen Weiterbildung sollte eine Neuorientierung während des Erwerbslebens mit dem Ziel, Fachkräfteengpässe zu verringern oder zu vermeiden, verstärkt gefördert werden. Hierzu muss die berufliche

Anpassungsfähigkeit und Mobilität durch Gelegenheiten zum Erlernen neuer Kenntnisse und Fähigkeiten – auch im Bereich der Soft Skills – verbessert werden. Die Förderung einer selbstbestimmten Weiterbildung jenseits berufsbezogener Qualifikationen, wie sie im Koalitionsvertrag mit dem Lebenschancen-BAföG vorgesehen ist, ist hierfür kein geeigneter Ansatz. Sie birgt das Risiko, dass Menschen an den Bedürfnissen des Arbeitsmarkts vorbei investieren. Vielversprechender ist das Instrument der Bildungsteilzeit nach österreichischem Vorbild, die eine Vereinbarung zwischen Arbeitgebenden und Beschäftigten voraussetzt und zudem der Bundesagentur für Arbeit Beratungs- und Kontrollfunktionen gibt. Auch sollte das bestehende Instrumentarium der Weiterbildungsförderung für Arbeitgebende erweitert werden, um Beschäftigten, die durch disruptive und transformative Prozesse gefährdet sind, ohne den Umweg über die Arbeitslosigkeit Brücken zu einer neuen Arbeitsstelle zu bauen (EFI, 2021).

Die Politik muss erst recht aktiv werden, wenn ihre Maßnahmen zur Bewältigung großer gesellschaftlicher Herausforderungen die Knappheiten am Arbeitsmarkt verstärken oder überhaupt erst auslösen. Darum benötigen etwa die Digitalisierungs- oder die Dekarbonisierungsstrategie der Bundesregierung eine damit im Einklang stehende und daraus abgeleitete Weiterbildungs- und Qualifizierungsstrategie. In der Nationalen Weiterbildungsstrategie wurden hierzu bereits einige richtige Schwerpunkte gesetzt (BMAS und BMBF, 2021). Letztlich können die in diesem Rahmen entwickelten und die vorhandenen Instrumente zur Unterstützung der beruflichen Weiterbildung nur dann breit in der Praxis wirken, wenn sie besser bekannt und noch leichter zugänglich werden.

## Bittere Medizin

Die unangenehme Wahrheit ist: Gegen die großen akuten Fachkräfteengpässe, die aus drei starken transformativen Prozessen – Digitalisierung, Dekarbonisierung, alternde Gesellschaft – gespeist und durch die gerade erlebte Zeitenwende noch einmal verstärkt werden, gibt es kaum simple Mittel. Ihre Überwindung wird dauern. Arbeitgebende, Bürger:innen und Politik müssen einiges mehr leisten. Sie werden um diese Zumutungen aber nicht herumkommen, wenn der Wohlstand in Deutschland nachhaltig gesichert werden soll.


Dr. Ulf Rinne ist Head of Scientific Management und Senior Research Associate am IZA — Institut zur Zukunft der Arbeit in Bonn.


Literatur

Bachmann R Jäger P Jessen R A Split Decision: Welche Auswirkungen hätte die Abschaffung des Ehegattensplittings auf das Arbeitsangebot und die Einkommensverteilung? Zeitschrift für Wirtschaftspolitik 2021 70 2 105 131 10.1515/zfwp-2021-2052
BGL — Bundesverband Güterkraftverkehr Logistik und Entsorgung (2021), Fahrermangel JETZT entgegenwirken! BGL stellt Aktionsplan Fahrermangel vor, Pressemitteilung, 7. Oktober.
Blömer, M., P. Brandt und A. Peichl (2021), Raus aus der Zweitverdienerinnenfalle: Reformvorschläge zum Abbau von Fehlanreizen im deutschen Steuer- und Sozialversicherungssystem, Bertelsmann Stiftung.
Blömer M Consiglio V Reform der Mini- und Midijobs: Verschärft die Ampel-Koalition die Teilzeitfalle? ifo Schnelldienst 2022 75 4 12 18
BMAS — Bundesministerium für Arbeit und Soziales und BMBF — Bundesministerium für Bildung und Forschung (2021), Umsetzungsbericht: Nationale Weiterbildungsstrategie.
Bonin, H. (2020), Fachkräftemangel in der Gesamtperspektive, in K. Jacobs, A. Kuhlmey, S. Greß, J. Klauber und A. Schwinger (Hrsg.), Pflege-Report 2019, Springer, 61–69.
Bonin, H., W. Eichhorst, A. Krause-Pilatus und U. Rinne (2021a), Wirksamkeitsanalyse der Corona-Maßnahmen, BMAS Forschungsbericht, 573.
Bonin, H., A. Krause-Pilatus und U. Rinne (2021b), Arbeitssituation und Belastungsempfinden von abhängig Beschäftigten im von der Corona-Pandemie geprägten Jahr 2021, BMAS Forschungsbericht, 570/10.
Bonin, H., W. Eichhorst, A. Krause-Pilatus und U. Rinne (2021c), Auswirkungen der Corona-Krise auf das Familien- und Erwerbsleben, BMAS Forschungsbericht, 574.
Deutscher Bundestag (2022), Bundeskanzler Olaf Scholz: Wir erleben eine Zeitenwende, https://www.bundestag.de/dokumente/textarchiv/2022/kw08-sondersitzung-882198 (23. August 2022).
EFI — Expertenkommission Forschung und Innovation (2021), EFI Gutachten 2021.
EFI — Expertenkommission Forschung und Innovation (2022), EFI Gutachten 2022.
Gleiser, P., S. Hensgen, C. Kagerl, U. Leber, D. Roth, J. Stegmeier und M. Umkehrer (2022), Knapp zwei Prozent der deutschen Betriebe haben bislang Geflüchtete aus der Ukraine eingestellt, IAB-Forum, 24. Juni, https://www.iab-forum.de/knapp-zwei-prozent-der-deutschenbetriebe-haben-bislang-gefluechtete-aus-der-ukraine-eingestellt/(23. August 2022).
IG Metall et al. (2022), Erfolgreiche Klimawende braucht leistungsfähiges Handwerk, https://www.zvshk.de/presse/medien-center/pressemit-teilungen/details/artikel/7631-gemeinsame-pressemitteilung/ (23. August 2022).
OECD (2017), Dare to Share — Deutschlands Weg zur Partnerschaftlichkeit in Familie und Beruf, https://www.oecd.org/els/dare-to-share-deutschlands-weg-zur-partnerschaftlichkeit-in-familie-und-beruf-9789264263420-de.htm (23. August 2022).
Röttger, C. und E. Weber (2022), Es gab keinen Big Quit in Deutschland, Ökonomenstimme, https://www.oekonomenstimme.org/artikel/2022/06/es-gab-keinen-big-quit-in-deutschland/ (23. August 2022).
Straubhaar T Kolev G Matthes J Flach L Teti F Görg H Kamin K Glassman A Wolff G B Globalisierung in der Krise? Wirtschaftsdienst 2021 101 11 840 861 10.1007/s10273-021-3040-0
Wippermann, C. (2012), Frauen im Minijob: Motive und (Fehl-)Anreize für die Aufnahme geringfügiger Beschäftigung im Lebensverlauf, https://www.bmfsfj.de/resource/blob/93862/4ba520100f0bde228598d1271c32cfd4/frauen-im-minijob-data.pdf (23. August 2022).
